# SARS-CoV-2 Infection and COVID-19 Vaccine Antibody Responses in Two Canadian Cohorts of Persons Living with HIV

**DOI:** 10.3390/antib15020030

**Published:** 2026-04-03

**Authors:** Sharon L. Walmsley, Leif Erik Lovblom, Bryan Boyachuk, Curtis Cooper, Valérie Martel-Laferrière, Mona Loutfy, Marie-Louise Vachon, Shariq Haider, Pamela Aldebes, Karen Colwill, Anne Claude Gingras, Freda Qi, Marina B. Klein

**Affiliations:** 1 Division of Infectious Diseases, University Health Network, Toronto, ON M5G 2C4, Canada; bryan.boyachuk@uhn.ca; 2 Faculty of Medicine, University of Toronto, Toronto, ON M5S 1A1, Canada; 3 Biostatistical Research Unit, University Health Network, Toronto, ON M5G 2C4, Canada; erik.lovblom@thebru.ca; 4 Ottawa Hospital Research Institute, Ottawa, ON K1Y 4E9, Canada; ccooper@ottawahospital.on.ca; 5 CHUM-Centre Hospitalier de l’Université de Montréal, Montreal, QC H2X 0A9, Canada; valerie.martel-laferriere.med@ssss.gouv.qc.ca; 6 Women’s College Hospital Research Institute, Toronto, ON M5G 1N8, Canada; 7 Faculty of Medicine, CHU de Québec-Université Laval, Quebec, QC G1V 4G2, Canada; 8 McMaster University Hospital, Hamilton, ON L8S 4K1, Canada; haider@mcmaster.ca; 9 The Research Institute of the McGill University Health Centre, Montreal, QC H3A 1A1, Canada; 10 Lunenfeld-Tanenbaum Research Institute, Mount Sinai Hospital, Toronto, ON M5G 1X5, Canada; 11 Department of Molecular Genetics, University of Toronto, Toronto, ON M5S 1A8, Canada; 12 Division of Infectious Diseases, McGill University Health Center, Montreal, QC H4A 3J1, Canada

**Keywords:** HIV, COVID-19, vaccines, antibody responses, hepatitis C, SARS-CoV-2

## Abstract

**Objectives:** To determine the incidence and outcomes of SARS-CoV-2 infection and to evaluate seroconversion rates and quantify antibody responses to COVID-19 vaccines in two cohorts of persons living with HIV at a possible higher risk of poor outcomes (HCV coinfection and those over the age of 65 years). **Methods:** We included participants from two established cohorts of persons living with HIV, those who were older than 65 years of age, and those with hepatitis C (HCV) co-infection. Four hundred and seventy-one participants completed questionnaires on SARS-CoV-2 infection and COVID-19 vaccine doses and submitted peripheral blood specimens for measuring antibody levels to COVID-19 antigens, full-length spike trimer, its receptor binding domain (RBD), and nucleocapsid protein (N) at 6-month intervals up to three visits between February 2021 and December 2024. Logistic and ordinal logistic regression models evaluated predictors of seroconversion and antibody levels. **Results:** Overall, 51% of participants developed a SARS-CoV-2 infection, but it was mild, with only nine requiring hospital admission and no deaths. Overall, 99% of tested specimens had antibodies above threshold to either spike or RBD proteins. Specimens that did not and those with lower antibody levels had testing earlier in the pandemic, and were from participants with fewer vaccine doses, and did not have natural infection. Age, depression, comorbidity, HCV co-infection, current substance use, CD4 count, or HIV viral load were predictive of antibody level. Those with hybrid immunity had higher antibody responses. **Conclusions:** In cohorts of persons with HIV-HCV coinfection and those who are ageing, we observed high rates of seroconversion to COVID-19 antigens. Antibody levels were higher among those with more vaccine doses, hybrid immunity, and later in the pandemic waves. Although 51% developed a breakthrough infection, outcomes were mild with no deaths.

## 1. Background

Persons living with HIV (human immunodeficiency virus), especially those with greater degrees of immunosuppression as indicated by lower CD4 cell counts and those with uncontrolled HIV replication, appear to have higher rates of poor outcomes of SARS-CoV-2 (severe acute respiratory syndrome coronavirus) infection [1]. There was concern that persons living with HIV would have a lower response to COVID-19 vaccines than was observed in the general population, similar to observations with other vaccines (i.e., against pneumococcus, influenza, or hepatitis B) [2,3,4].

Serological assays for COVID-19 typically use the highly immunogenic nucleocapsid and spike proteins of SARS-CoV-2 as antigens [5,6]. Nucleocapsid is a highly abundant structural protein involved in viral RNA packaging and core assembly. In contrast, the trimeric spike protein is expressed on the viral surface, where it mediates viral entry through binding of its receptor-binding domain (RBD) to the host angiotensin-converting enzyme 2 (ACE2) receptor [7]. Spike is the primary target of neutralizing antibodies, and all vaccines approved in Canada encode the spike antigen only [8,9]. Consequently, seroreactivity to nucleocapsid indicates a prior infection, whereas antibodies to spike or RBD signal may arise from vaccination and/or a prior infection.

The data regarding immunogenicity to COVID-19 vaccines is varied. A systematic review [10] concluded that vaccine-induced seroconversion in persons living with HIV is high, with rates over 70%, but lower in those with lower CD4 counts and higher viral loads [11,12,13,14,15]. The majority of studies have reported on middle-aged white men with well-controlled HIV [16]. The data is also difficult to compare across studies and is confounded by additional patient demographic characteristics such as age, race, and comorbidity. The seroconversion rates could also vary by the vaccine type [17,18], number of vaccine doses, intervals between doses [19], the time from vaccine to antibody testing, the presence of hybrid immunity, the specific assay, and the cut-off values used [20,21,22].

Despite good results overall, less is known about vaccine responses in persons with HIV who might be at increased risk of poor outcomes, such as those ageing with HIV with underlying comorbidity or those with hepatitis C (HCV) coinfection who may have active substance use and/or depression. The purpose of this study was to determine the rates of seroconversion, the levels of binding antibody to COVID-19 antigens, and the frequency and severity of SARS-CoV-2 infection in these sub-populations of persons with HIV.

## 2. Methods

To address these questions, we chose to assess SARS-CoV-2 infection and response to COVID-19 vaccines in participants of two ongoing national Canadian longitudinal cohort studies of persons living with HIV. We selected these cohorts for study because of potential risk factors for higher rates of SARS-CoV-2 infection or poorer vaccine responses. The published objectives and demographics of these cohorts are available [23,24]. Briefly, the Canadian Trials Network study-CTN 222 (https://cocostudy.ca/; accessed on 30 March 2026) has longitudinally collected data on persons with HIV and HCV coinfection since 2003 with the goal of evaluating the impact of various social and demographic factors and therapies on liver disease outcomes and mortality [25]. In previous work, we have demonstrated that the rates of previous (81%) or active injection drug use (34%) and depression are high (58% had a CES-10 (Centres for Epidemiologic studies depression scale-10) score greater than 10% [23,26]. CTN 314 (CHANGE HIV) (https://www.changehivstudy.com/; accessed on 30 March 2026), established in 2019, enrolled persons over 65 years of age and is evaluating factors that contribute to healthy ageing with HIV [24].

With the onset of the COVID-19 pandemic, the parent CTN 222 and CTN 314 studies pivoted to an online data collection method and questionnaires for follow-up. Both parent studies had prior approval by their local REB and the CTN community advisory committees and received amendment approval for the transition to online assessment. At the time of the transition, this optional COVID-19 sub-study was added and approved by the University Health Network (UHN) Research Ethics Board (REB) (CAPCR # 20-5986) and the REB of participating sites.

With this latter approval, active participants of the two parent cohorts were approached at their main study visit for enrollment into this COVID-19 sub-study. With written consent, they completed a questionnaire about SARS-CoV-2 infection and its outcome, and information on the receipt of COVID-19 vaccines and booster doses, and submitted a venipuncture blood specimen for the determination of antibody levels. The activities were planned to be repeated at 6-month intervals for up to three visits.

Seven of nineteen active CTN 222 sites and 2/6 active CTN 314 sites were able to participate in this substudy. Consent was obtained from a questionnaire that was completed by 471 participants, including 51% of active participants from the contributing CTN 314 sites and 76% of active participants in the contributing CTN 222 sites.

Key demographic and clinical variables collected included age, gender, racial/ethnic background, annual income, duration of HIV infection, comorbidities, alcohol use, self-reported presence of depressive symptoms, and self-reported use of substances such as marijuana, cocaine/heroin, opioids, other injectable drugs, and other oral or inhaled substances used at the time of the questionnaire. Clinical measures, including HIV viral load and CD4 cell count, were extracted from the participant’s medical record at the time of the sub-study enrollment.

### 2.1. Laboratory Studies

Serological Assays: Blood specimens were shipped to the University Health Network research unit, registered in a RED Cap (Research Electronic Data Capture) database, and transferred to the Lunenfeld-Tanenbaum Research Institute (LTRI) at Sinai Health for processing (Sinai REB study 23-0120-E). A chemiluminescent ELISA assay was used to detect antibody levels to full-length spike trimer, its receptor binding domain (RBD), and nucleocapsid protein (N) as previously described. Specimens were tested by Enzyme Linked Immunosorbent Assay (ELISA) for antibodies (IgG) against the SARS-CoV-2 full-length spike trimer, its receptor binding domain (RBD), and nucleocapsid protein (N) as previously described [27,28]. The ELISA developed in-house on serum and plasma was optimized for sensitivity and specificity parameters. As samples with high antibody levels saturate the assays, preventing accurate measurement, all samples were tested at two dilutions (primary dilution of 2.5 µL/well (1:4) and a secondary dilution of 0.156 µL/well (1:64)) to ensure a large fraction of the measurements would be tested within the linear range of quantification. We selected to profile total IgG antibodies to the indicated antigens since our results and others show a strong correlation, especially between anti-RBD IgG levels and neutralization titers, enabling us to infer neutralization changes.

Briefly, LUMITRAC 600 high-binding white polystyrene 384-well microplates (#781074, Greiner Bio-One, Monroe, NC, USA; #82051-268, VWR, Radnor, PA, USA) were pre-coated overnight with 10 µL per well of antigen (Ag): 50 ng spike, 20 ng RBD (331–521), and 7 ng N, all supplied by the National Research Council of Canada (NRC). Plates were centrifuged at 1000 rpm for 1 min to ensure even coating, then incubated overnight at 4 °C. The next day, the assay was performed at room temperature with washing twice in 115 µL/well PBS-T before each of the following four steps. Step one: wells were blocked for 1 h in 115 µL 5% Blocker BLOTTO (#37530, Thermo Fisher Scientific, Waltham, MA, USA). Step two: 10 µL of serum diluted 1:160 or 1:2560 in 1% final Blocker BLOTTO in PBS-T was added and incubated for 2 h. Step three: 10 µL of an anti-human IgG fused to HRP (IgG#5 by NRC, 0.9 ng/well) diluted in 1% final Blocker BLOTTO in PBS-T was added, followed by a 1 h incubation. Step four: 10 µL of ELISA Pico Chemiluminescent Substrate (Thermo Fisher Scientific, #37069, diluted 1:4 in MilliQ distilled H_2_O) was added, and the plates were centrifuged at 1000 rpm for 1 min. After a 5 min incubation period, chemiluminescence signals were read on an EnVision 2105 Multimode Plate Reader (Perkin Elmer, Shelton, CT, USA) plate reader at 100 ms/well using an ultra-sensitive detector.

Raw chemiluminescent values were normalized to a synthetic standard included on each assay plate (VHH72-Fc supplied by NRC for spike/RBD or an anti-N IgG Ab from Genscript, #A02039, Piscataway, NJ, USA), to create relative ratios. Relative ratios with the linear range of the assay were further converted to binding Ab units (BAU/mL) using the WHO International Standard 20/136 as the calibrant, as previously described [27]. Positivity thresholds were determined for the 1:160 dilution using 3 standard deviations from the mean of control samples as previously described.

The vaccines secured by the Canadian government are spike-based, so reactivity to nucleocapsid should only be from natural infection. Monitoring anti-N antibodies helps to identify possible new infections or reinfections. The antibody threshold values used for positivity were 34.46 BAU/mL (binding antibody units) for N, 30.97 BAU/mL for RBD, and 11.28 BAU/mL for spike [29].

### 2.2. Statistical Methods

Descriptive statistics were calculated as median (Q1–Q3) and counts (%); between-group comparisons of demographic profiles were made using the Wilcoxon rank sum test (for continuous variables), the chi-square test (for categorical variables), or Fisher’s exact test (for categorical variables with cell counts of fewer than 5). Violin plots were used to display the distribution of antibody levels. They are a form of histogram in which each value is depicted by a dot; the wider the curve, the higher the frequency of values at that level. The median value is displayed by a solid horizontal line.

We identified factors associated with RBD levels in two different ways: first, we ran logistic regression models with the dichotomous outcome being RBD level below the threshold of detectability; second, we ran ordinal logistic regression models with the ordinal outcome being quartiles of RBD values (in increasing order). To account for the possibility of correlated within-person observations, generalized estimating equations were used to fit the models. Ordinal logistic regression was used for the continuous RBD outcome due to severe skew of the distribution; the proportional odds assumption was deemed to be sufficient for the data. For the logistic regression analysis, since the number of outcomes was low, only univariable models were run. On the other hand, an adjusted multivariable model was run for the ordinal logistic regression; for this model, we removed the observations with no vaccine history. An alpha level of 0.05 was used for statistical hypothesis tests.

Analyses were performed using R software, version 4.5.1; the package ‘ggplot2’ (version 4.0.1) was used for graphical displays, while the packages ‘geepack’ (version 1.3.12; logistic regression) and ‘multgee’ (version 1.9.0; ordinal logistic regression) were used for the statistical modelling.

## 3. Results

Overall, 471 participants consented to this sub-study. The first patient visit was on 10 February 2021, the last enrollment was on 20 April 2023, and the last follow-up visit occurred on 2 December 2024. Given the challenges of study visits during a pandemic, we did not have a complete data set as not all participants completed all questionnaires, nor all blood samples, nor were the data always collected at the same time points. We elected to include participants in the analysis if they had submitted at least one questionnaire and at least one blood specimen (*n* = 384), including 240 participants from CTN 222 (HIV-HCV co-infection) and 144 from CTN 314 (ageing). We excluded 86 who did not provide a blood specimen and one person who did not provide a questionnaire. Of the 384 included participants, 60 submitted one, 93 submitted two, and 231 submitted three blood specimens for the evaluation of SARS-CoV-2 antibodies (total of 939 values).

As blood samples were submitted at different time points for different participants, we elected to evaluate the antibody levels based on calendar times during different waves of the COVID-19 pandemic in Canada. We defined wave 1 as February to October 2021, when the alpha, beta, gamma, and delta variants of SARS-CoV-2 were circulating (*n* = 74 specimens). We defined wave 2 from October 2021 to November 2022 during the initial Omicron circulating period (*n* = 473 specimens). We defined wave 3 as after November 2023 during the circulating period for Omicron XBB 1.5 and other subvariants (*n* = 392 specimens).

The demographic profile of those included in the analysis is outlined in Table 1. The median age was 53 years. The majority were male, 84%; White, 82%; and had been living with HIV for a median of 24 years. Overall, 70% of the participants had an annual income of <$50K Canadian. Alcohol use was reported in 62%, marijuana in 44%, and other substances in a minority. Most (92%) had suppressed HIV viral replication (<50 copies/mL), and 6% had CD4 counts < 200 copies/mm^3^. As we would have anticipated from the inclusion criteria of the main studies, there are significant differences in the characteristics of the participants of the two cohorts. Further, there is some overlap as some persons with HCV coinfection were also over age 65 years, and some of the older cohort also had HCV. For the analysis of outcomes, we combined the cohorts and addressed the individual covariates.

### 3.1. SARS-CoV-2 Infection

The proportion of individuals who developed a SARS-CoV-2 infection increased over time, as determined either by self-report or by the determination of antibodies to N above threshold (Table 2). Overall, 36% of the tested specimens had antibodies to N–12% in wave 1, increasing to 31% and 47% in waves 2 and 3, respectively. Of the participants, 51% had a positive N above threshold on at least one blood specimen. This was consistent with the self-report, where 24% reported at least one SARS-CoV-2 infection, 3% during wave 1, and 15 and 30% respectively by waves 2 and 3.

Of the entire cohort, 11 were assessed in the hospital for their SARS-CoV-2 infection, 9 were admitted (2 from CTN 314, 7 from CTN 222), 4 required ICU care (all from CTN 222), and one person was intubated. There were no deaths related to the SARS-CoV-2 infection.

### 3.2. Vaccine Uptake and Serologic Response

Vaccine uptake in the population was high, with 371 (97%) reporting receipt of at least one vaccine dose over the course of the study, and 255 (74%) reporting at least 3 doses.

Overall, the majority of the samples showed evidence of seroconversion as demonstrated by antibody levels to RBD and spike above threshold. Overall, 5% of samples did not show seroconversion to RBD—27% during the first wave and 4% and 3% respectively for the second and third waves. Similarly, 2% of samples did not show seroconversion to spike-15% during wave 1, decreasing to 2% and 1% during waves 2 and 3 (Table 2). There were 34 participants who received two non-mRNA (modified adenovirus vector) vaccines for the initial vaccine series. All of these participants did seroconvert.

**Figure 1:**
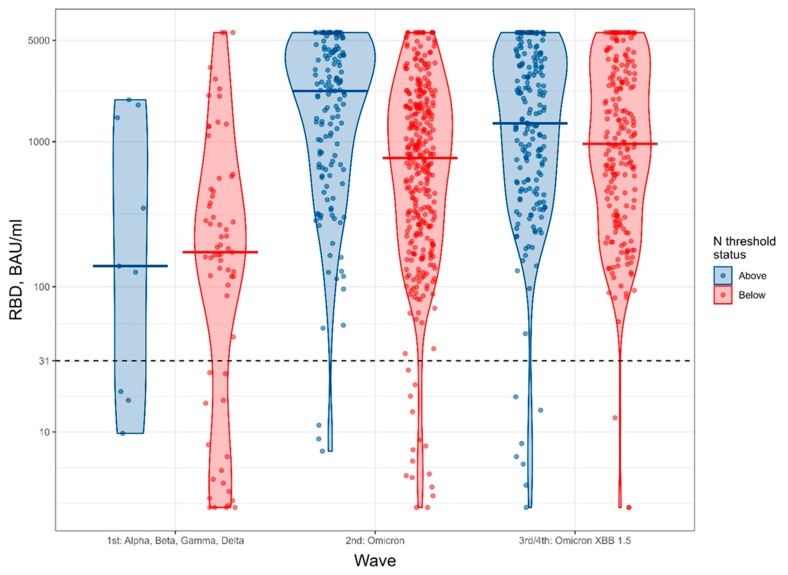
Violin plots of RBD levels according to wave and N status at time of measurement. Solid lines represent the median values; the dashed horizontal line represents the RBD threshold value. RBD-receptor binding domain. N-nucleocapsid.

The mean (range) of antibody levels with time is shown in Figure 1 and Table 2. Overall, the mean antibody level increased from the first to the subsequent waves after participants had received the first two doses of vaccine. In those two waves, the antibody levels to RBD and spike were higher in those who were also N positive above threshold, suggesting the presence of hybrid immunity (vaccine immunity plus immunity from natural infection).

We evaluated, using logistic regression, the factors associated with failure to seroconvert using RBD antibody levels below threshold as the outcome measure (Table 3). In univariable models, the odds of not seroconverting were lower after the first wave, lower if also N positive, and much lower if more than two vaccines were received. We did not find the presence of comorbidity (chronic heart or lung disease, diabetes, hypertension, cancer, HCV coinfection) nor active substance use or self-reported depression to be associated with lower seroconversion rates.

We also assessed, using logistic regression, factors associated with higher antibody levels to RBD (Table 4). In this analysis, we divided the cohort into quartiles with the lowest antibody value of 3.02 and the highest 5651 (BAU/mL). In the multivariable model, the odds of being in the lowest RBD quartile included testing during the first wave and the participant having less than two vaccine doses. Again, age, the presence of cancer, diabetes, and active substance use were not associated with lower antibody titres.

## 4. Discussion

In our two cohorts of persons living with HIV, those with HCV co-infection and those over the age of 65 years, an increasing proportion developed a SARS-CoV-2 infection over the course of our study. There were similar rates between self-report of an infection and the presence of antibody to N on laboratory testing. The higher percentage on laboratory testing could be due to asymptomatic infection or because persons did not test with symptoms. By the end of December 2024, half of the cohort had a natural infection, with most infections occurring during the Omicron waves despite high rates of vaccine uptake. These proportions are consistent with those of the general population of Canada, where the peak rate of infections occurred in 2022, with an estimated 75% of the population infected by 15 March 2023 [30]. Rates are also consistent with those of our decentralized study of COVID-19 in the general population of Ontario (STOPCoV), where 61% of those aged 30–50 and 57% of those aged >70 had at least one infection [29,31]. Although COVID-19 vaccines can modulate infection [32,33], they do not completely protect likely due to sub-optimal vaccine-induced seroprotection [34], waning immunity [35], and immune escape of new variants from the vaccine components [36,37]. The outcome of the SARS-CoV-2 infections in the current study was mild, with only nine persons (1.9%) requiring hospitalization, four (0.8%) requiring ICU care, and no deaths.

Vaccine uptake in our cohort was high. Early in the pandemic, there was some concern about the safety of the vaccine in those living with HIV. However, as more data emerged, safety was similar to that of the general population [18,38]. As those living with HIV were concerned about the risks and severity of infection, this led to high uptake rates [39]. Uptake rates in other populations of persons living with HIV in Ontario and Canada were also high [40,41].

We found good antibody responses to SARS-CoV-2 antigens with less than 5% failing to seroconvert on any one specimen submitted. In the published systematic review [10] 99.2% had detectable antibody to spike, but the included studies did not always differentiate between those vaccinated and those who had detectable antibody due to a natural infection. In our cohort, failure to seroconvert was higher in the earlier waves of infection and in those with fewer vaccine doses. We did not find age, comorbidity (diabetes, cancer, HCV co-infection, chronic heart or lung disease, hypertension, CD4 count, or viral load to be a significant factor, as shown in other studies. Similarly, we did not find active substance use or self-reported depression to be related to lower seroconversion rates.

The optimal correlates for protection against SARS-CoV-2 infections are unknown. High levels of neutralizing antibodies against RBD are the strongest predictor of protection from symptomatic infection [34,42]. Serum anti-spike and anti-RBD IgG levels are thought to represent reliable proxy markers for infection [43]. However, anti-S and anti-RBD IgA in the mucosal surfaces [44] and cellular immunity are likely also contributory [31,45]. Studies have shown there is increased protection with higher levels of binding and neutralizing antibodies; however, there is no universal confirmed protective threshold [31,46,47,48]. We studied predictors of quantitative antibody responses. We found that the levels increased after the first COVID-19 wave, likely in response to participants having received the initial two-dose vaccine series. The levels then remained high during the subsequent waves of infection and were higher in those who were also N positive, indicative of hybrid immunity and boosting of antibody levels. We divided the responses into quartiles. Those samples with antibodies in the lowest quartile were more likely to be tested in the early phase, and from participants who had received fewer vaccine doses.

In the SCAPE study conducted in the United Kingdom [49], a high rate of seroconversion was demonstrated, with 99.2% of 520 persons having antibody levels above the threshold. However, in this study, age over 60 years was associated with lower antibody levels to spike. In contrast, in our cohort, the probability of being in the lower RBD category was not associated with age.

The reported literature is inconsistent as to rates of seroconversion and antibody levels in those with lower CD4 cell counts or uncontrolled HIV infection. As in other studies, despite advancing age and/or HCV coinfection, the majority of our participants had CD4 counts > 200/mm^3^ and controlled viral replication [10]. In our statistical models, we did not find either variable associated with failure to seroconvert or with lower levels of RBD antibody response. In a Brazilian study [50], lower antibody levels were found in those with CD4 counts less than 200/mm^3^, but this was only significant in the individuals with 2 doses of vaccine and not for those with 3 or 4 doses, suggesting that lower antibody responses can be overcome with additional vaccine doses. Although they found higher levels in those with suppressed HIV viral RNA, they did not consider the number of vaccine doses in this sub-analysis.

To date, there has not been a protective level of antibodies to prevent SARS-CoV-2 infection, and in our study, breakthrough infections occurred despite vaccination. Further, the development of hybrid immunity resulted in increased antibody levels. This adds further to the observation that immune escape can occur with the emerging variants and highlights the need for continued boosters matching the circulating strains [51,52,53].

Despite our cohorts having a potential increased risk of lower vaccine responses as they consisted of ageing persons with comorbidity and those with active substance use and depression, we still identified potent antibody responses to SARS-CoV-2. In a study from the United States [54], evaluating racial and ethnic minorities with HIV and those with substance use and comorbidities, the authors described demographic differences between vaccinated and unvaccinated participants. Although in their adjusted models, CD4 < 200/mm^3^ and VL 200–5000 copies/mL were associated with lower IgG titres in those who had received 2 doses of vaccine, they did not take into consideration the impact that hybrid immunity might have on these values. Other studies have shown lower antibody responses in those with comorbidities, especially diabetes, hypertension, and obesity [49]. In our study, we did not find a difference in antibody response in those reporting no comorbidity relative to those reporting cancer, diabetes, or liver disease.

A limitation of this study is that we did not have a complete data set with all participants completing questionnaires and submitting blood specimens at the same time and at standardized times after the vaccine. However, we compensated for this by assessing the results of the antibody levels at calendar times during the COVID-19 waves in Canada. We also relied on self-reports of SARS-CoV-2 infections and vaccine doses that could be subject to recall bias. We did not find that higher CD4 counts or HIV RNA suppression were predictive of seroconversion or antibody levels. Although this could imply vaccine boosters and natural infection compensated by increasing antibody levels, we acknowledge that the majority of our participants were suppressed and had good CD4 cell counts. We also acknowledge that binding antibodies may not be the best predictors of protection from infection. There were some differences not only in the number but also in the brand of vaccines that were used. However, all participants who received non-mRNA vaccines as the initial series did seroconvert. A strength of our study is that we demonstrated high antibody levels in cohorts of HIV patients who could be compromised by age, comorbid illness, HCV coinfection, depression, or substance use issues. We demonstrated that the levels of antibodies remained high over time. Our models included factors that could impact antibody levels, including the number of vaccine doses and hybrid immunity, in addition to HIV-related factors.

## 5. Conclusions

We conclude that persons with HIV-HCV co-infection and those who are ageing with HIV mount good antibody responses to COVID-19 vaccines. More vaccine doses and the presence of hybrid immunity result in higher antibody levels. Despite high and sustained antibody levels, a significant proportion of our cohorts did acquire a natural infection during the Omicron wave, likely because of a mismatch between vaccine and circulating strain. Nonetheless, the vaccines may have modulated the infection as they were generally mild with few hospitalizations and no deaths. Our data support the benefit of COVID-19 vaccines in persons with HIV.

## Figures and Tables

**Table 1:** Demographic profile of the study participants.

Characteristic	Full Sample (*n* = 384)	CTN222 HIV/HCV (*n* = 240)	CTN314 Ageing (*n* = 144)	*p*-Value
Age, years	53 (44–68)	46 (39–51)	70 (67–73)	<0.001
Years living with HIV	24 (17–30)	22 (16–28)	28 (21–32)	<0.001
Male gender	322 (84%)	190 (79%)	132 (92%)	0.002
Race				<0.001
Indigenous	13 (3%)	13 (5%)	0 (0%)	
Asian	9 (2%)	4 (2%)	5 (3%)	
Black	22 (6%)	10 (4%)	12 (8%)	
Caucasian	314 (82%)	203 (85%)	111 (77%)	
Hispanic	15 (4%)	9 (4%)	6 (4%)	
Other	11 (3%)	1 (0%)	10 (7%)	
Income (Can $)				<0.001
<20K	38 (10%)	15 (6%)	23 (16%)	
20K–49.9K	232 (60%)	182 (76%)	50 (35%)	
50K–99.9K	67 (17%)	27 (11%)	40 (28%)	
>100K	44 (11%)	13 (5%)	31 (22%)	
Current Substance Use				
Alcohol	219 (62%)	139 (59%)	80 (70%)	0.049
Marijuana	169 (44%)	127 (53%)	42 (30%)	<0.001
Cocaine or heroin	78 (20%)	78 (33%)	0 (0%)	<0.001
Opioids	27 (7%)	18 (8%)	9 (6%)	0.81
Other injectable agents	8 (2%)	5 (2%)	3 (2%)	0.99
Other oral inhaled agents	14 (4%)	11 (5%)	3 (2%)	0.33
Viral load not detected (<50 copies/mL)	336 (92%)	210 (88%)	126 (99%)	<0.001
CD4 count/mm^3^				0.039
<200	20 (6%)	16 (7%)	4 (3%)	
200–500	126 (35%)	72 (31%)	54 (44%)	
>500	209 (59%)	143 (62%)	66 (53%)	
Current Depression	131 (34%)	109 (45%)	22 (15%)	<0.001
Comorbidities				
Chronic heart disease	53 (14%)	29 (12%)	24 (17%)	0.27
Chronic lung disease	91 (24%)	73 (30%)	18 (12%)	<0.001
High blood pressure	105 (27%)	50 (21%)	55 (38%)	<0.001
Cancer	36 (9%)	9 (4%)	27 (19%)	<0.001
Diabetes	56 (15%)	28 (12%)	28 (19%)	0.052
Hepatitis C	265 (69%)	240 (100%)	4 (3%)	<0.001
None of the above	42 (11%)	0 (0%)	42 (29%)	<0.001

Data described as median (Q1–Q3) or as *n*(%). CTN-Canadian trials network.

**Table 2:** Descriptive statistics of each lab value, with the corresponding self-reported infection and vaccine history, by wave.

Characteristic	Wave 1 (*n* = 74)	Wave 2 (*n* = 473)	Wave 3 (*n* = 392)
Previous self-reported positive COVID-19 test	2 (3%)	79 (17%)	131 (36%)
Antibody to Nucleocapsid above threshold	9 (12%)	145 (31%)	185 (47%)
Antibody to Nucleocapsid (BAU/mL)	6.2 (3.5–11.4)	10.3 (4.1–59.5)	26.8 (7.1–103.8)
Number of vaccine doses at time of testing			
0	10 (14%)	13 (3%)	11 (3%)
1 or 2	62 (86%)	227 (50%)	79 (22%)
>2	0 (0%)	217 (47%)	264 (75%)
Antibody to RBD below threshold	20 (27%)	18 (4%)	10 (3%)
Antibody to RBD (BAU/mL)	170.1 (25.3–536.9)	969.5 (307.4–2512.7)	1167.2 (403.8–3252.6)
Antibody to Spike below threshold	11 (15%)	8 (2%)	4 (1%)
Antibody to Spike (BAU/mL)	205.7 (70.4–1099.7)	1375.6 (523.6–1893.7)	1461.7 (635.8–1893.7)

Data described as median (Q1–Q3) or as *n* (%). BAU–binding antibody units. RBD-receptor binding domain.

**Table 3:** Results of the univariable logistic regression models for RBD levels being below the threshold.

Covariate	Odds Ratio (95% CI)	*p*-Value
Wave		
Wave 1	(Reference)	
Wave 2	0.09 (0.05, 0.16)	<0.001
Wave 3	0.06 (0.03, 0.13)	<0.001
Age, per 1 year	1.00 (0.98, 1.03)	0.86
Undetectable viral load (<50 copies/mL)	1.05 (0.24, 4.64)	0.95
CD4 count/mm^3^		
<200	(Reference)	
200–500	4.87 (0.66, 36.20)	0.12
>500	1.94 (0.25, 14.93)	0.52
Any substance use (excluding alcohol)	1.45 (0.68, 3.09)	0.33
Current Depression	0.97 (0.42, 2.23)	0.94
Comorbidities		
Chronic heart disease	1.25 (0.47, 3.31)	0.65
Chronic lung disease	0.64 (0.26, 1.56)	0.33
High blood pressure	0.53 (0.18, 1.55)	0.25
Cancer	0.76 (0.12, 4.78)	0.77
Diabetes	0.65 (0.22, 1.90)	0.43
Hepatitis C	1.10 (0.54, 2.21)	0.80
None of the above	0.78 (0.22, 2.73)	0.70
Antibody to Nucleocapsid above threshold	0.50 (0.25, 0.97)	0.041
Number of previous vaccines		
0	(Reference)	
1 or 2	0.02 (0.01, 0.07)	<0.001
>2	0.001 (0, 0.008)	<0.001

RBD-receptor binding domain.

**Table 4:** Results of the multivariable ordinal logistic regression model for RBD level quartile, with the odds ratio representing the odds of being in a lower quartile.

Covariate	Adjusted Odds Ratio (95% CI)	*p*-Value
Wave		
Wave 1	(Reference)	
Wave 2	0.34 (0.19, 0.62)	0.001
Wave 3	0.38 (0.20, 0.73)	0.003
Age, per 1 year	1.00 (0.98, 1.02)	0.88
Undetectable viral load (<50 copies/mL)	0.65 (0.34, 1.22)	0.18
Current substance use (exclude alcohol)	1.21 (0.88, 1.66)	0.24
Cancer	0.74 (0.44, 1.26)	0.26
Diabetes	0.80 (0.51, 1.26)	0.33
Hepatitis C	1.27 (0.74, 2.19)	0.39
Antibody to Nucleocapsid above threshold	0.32 (0.24, 0.43)	<0.001
Number of vaccines at time of testing		
0	(Removed)	
1 or 2	(Reference)	
>2	0.45 (0.34, 0.61)	<0.001

RBD-receptor binding domain.

## Data Availability

The datasets are available with a reasonable request from the corresponding author.

## Abbreviations

The following abbreviations are used in this manuscript:

ACE2	Angiotensin-converting enzyme 2.
BAU	Binding antibody units.
CES-10	Centres for Epidemiologic studies depression scale-10.
CTN	Canadian HIV Trials Network.
ELISA	Enzyme-linked immunosorbent assay.
LTRI	Lunenfeld-Tanenbaum Research Institute.
N	Nucleocapsid protein.
NRC	National Research Council of Canada.
RBD	Receptor-binding domain.
UHN	University Health Network.
